# Non-invasive diagnosis strategy of hepatocellular carcinoma in low-risk population

**DOI:** 10.1186/s12885-022-09812-w

**Published:** 2022-06-28

**Authors:** Zonglin Xie, Zhenpeng Peng, Yujian Zou, Han Xiao, Bin Li, Qian Zhou, Shuling Chen, Lixia Xu, Jingxian Shen, Yunxian Mo, Sui Peng, Ming Kuang, Jianting Long, Shi-Ting Feng

**Affiliations:** 1 Department of Gastroenterology and Hepatology, The First Affiliated Hospital of Sun Yat-Sen University, Guangzhou, China; 2 Department of Radiology, the First Affiliated Hospital, Sun Yat-Sen University, Yuexiu Distinct, 58 Zhongshan Road 2, Guangzhou, 500018 China; 3 Department of Radiology, The Affiliated Dongguan Hospital, Southern Medical University, Dongguan, China; 4 Division of Interventional Ultrasound, The First Affiliated Hospital of Sun Yat-Sen University, Guangzhou, China; 5 Clinical Trial Unit, The First Affiliated Hospital of Sun Yat-Sen University, Guangzhou, China; 6 Department of Liver Surgery, The First Affiliated Hospital of Sun Yat-Sen University, Guangzhou, China; 7 Department of Oncology, the First Affiliated Hospital, Sun Yat-Sen University, Yuexiu Distinct, 58 Zhongshan Road 2, Guangzhou, 500018 China; 8 Department of Medical Imaging, Sun Yat-Sen University Cancer CenterState Key Laboratory of Oncology in South ChinaCollaborative Innovation Center for Cancer Medicine, Guangzhou, China

**Keywords:** Hepatocellular carcinoma, LI-RADS, Diagnosis, Low-risk, Non-invasive

## Abstract

**Aims:**

With prevalence of hepatocellular carcinoma (HCC) in low-risk population (LRP), establishing a non-invasive diagnostic strategy becomes increasingly urgent to spare unnecessary biopsies in this population. The purposes of this study were to find characterisics of HCC and to establish a proper non-invasive method to diagnose HCC in LRP.

**Methods:**

A total of 681 patients in LRP (defined as the population without cirrhosis, chronic HBV infection or HCC history) were collected from 2 institutions. The images of computed tomography (CT) and magnetic resonance imaging (MRI) were manually analysed. We divided the patients into the training cohort (*n* = 324) and the internal validating cohort (*n* = 139) by admission time in the first institution. The cohort in the second institution was viewed as the external validation (*n* = 218). A multivariate logistic regression model incorporating both imaging and clinical independent risk predictors was developed. C-statistics was used to evaluate the diagnostic performance.

**Results:**

Besides the major imaging features of HCC (non-rim enhancement, washout and enhancing capsule), tumor necrosis or severe ischemia (TNSI) on imaging and two clinical characteristics (gender and alpha fetoprotein) were also independently associated with HCC diagnosis (all *P* < 0.01). A clinical model (including 3 major features, TNSI, gender and AFP) was built to diagnose HCC and achieved good diagnostic performance (area under curve values were 0.954 in the training cohort, 0.931 in the internal validation cohort and 0.902 in the external cohort).

**Conclusions:**

The clinical model in this study developed a satisfied non-invasive diagnostic performance for HCC in LRP.

**Supplementary Information:**

The online version contains supplementary material available at 10.1186/s12885-022-09812-w.

## Introduction

For the population with high-risk factors (HRP) of hepatocellular carcinoma (HCC), including cirrhosis, chronic hepatitis B (CHB) and HCC history, the diagnosis of HCC could rely on imaging. However, patients with liver nodule but without any high-risk factors of HCC are commonly observed clinically. The diagnostic strategy for these low-risk population (LRP, people without high-risk factors of HCC) is still limited due to scant evidence [1]. Recently, what makes the dilemma more urgent is that the proportion of HCC patients belonging to LRP has fueled rapidly for the prevalence of obesity, metabolic syndrome and non-alcoholic fatty liver disease (NAFLD) [2, 3]. According to the current guidelines, invasive liver biopsy is indispensable for the LRP to diagnose HCC, which is limited by high false negative rate and many complications such as bleeding and needle implantation [4]. Therefore, finding an accurate non-invasive diagnostic approach could avoid repeated invasive confirmation and unnecessary follow-up.

Currently, imaging features of dynamic enhanced CT/MRI can be effectively utilised to characterise liver nodules. The major imaging features of HCC, arterial hyperenhancement (APHE) followed by wash-out (WO), have been widely verified that the pretest probability of HCC is sufficiently high and the pre-test probability of nodules mimicking HCC is sufficiently low in HRP [5]. Although a few of non-HCC liver nodules, such as intrahepatic cholangiocarcinoma (iCCA), liver adenoma, angiomyolipoma, and inflammatory pseudotumor are identical to HCC on imaging, higher prevalence of these non-HCC nodules in LRP impaired diagnostic specificity[6, 7]. Moreover, HCCs in LRP are prone to large size with central necrosis and infiltrative performance, promoting atypical appearance on imaging [8, 9]. To establish an appropriate non-invasive diagnostic approach of HCC in LRP, multi-dimensional consideration might be necessary.

Previous studies reported that ancillary features could be applied optionally once the imaging diagnosis of liver nodules cannot be confirmed in HRP [10, 11]. Alpha fetal protein (AFP), as a well-known clinical parameter, is closely associated with HCC development with high diagnostic specificity. Spontaneous intra-nodule necrosis, as an ancillary imaging feature, could be found in many types of solid tumors including HCC [12]. It usually suggests a poorly differentiated neoplasm that has overloaded its blood supply, predicting a poor prognosis and an increased metastatic potential [13]. These facts show that ancillary features could play a vital role for diagnosis of HCC in LRP.

In this study, we analysed the imaging features and clinical information of LRP to develop a non-invasive diagnostic procedure of HCC with high accuracy to spare the unnecessary biopsies in LRP.

## Methods

### Study cohort

**Fig. 1:**
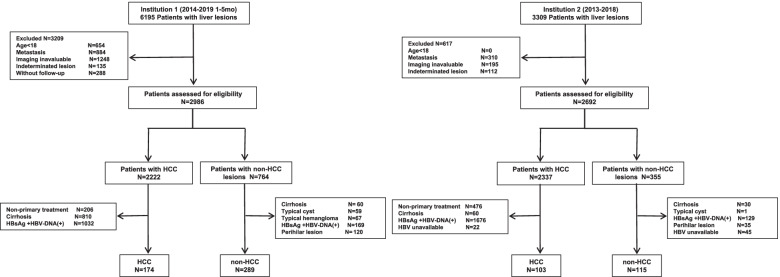
Flow chart outlining patient selection and grouping process in both institutions

Two cohorts of patients with liver nodules were consecutively collected from the First Affiliated Hospital of Sun Yat-sen university (the 1st institution) between Jan 2014 to May 2019 and Sun Yat-sen University Cancer Center (the 2nd institution) between Jan 2013 to Dec 2018. Inclusion criteria for enrollment were as follows. (I) adults in LRP. (II) available dynamic enhanced CT/MRI imaging data before treatment. (III) conclusive pathological diagnosis of the liver nodule. (IV) no more than 12-week interval between the final pathological diagnosis and imaging examination. Exclusion criteria were as follows. (I) histological diagnosis of liver metastases. (II) definite benign nodules that biopsy was considered unfeasible or not necessary (e.g., typical cysts, hemangioma). (III) perihilar nodules beyond the liver parenchyma. The collection flowcharts of the two cohorts were shown in Fig. 1. The Ethics Committee of the First Affiliated Hospital of Sun Yat-sen university approved this study and waived the requirement for informed consent.

HRP was defined based on the Liver Imaging Reporting and Data System (LI-RADS). Specifically, high-risk factors of HCC were defined as cirrhosis, current chronic hepatitis B virus (HBV) infection, current or prior HCC. LRP was defined as people with none of the above risk factors (e.g., non-cirrhotic HCV, non-cirrhotic NAFLD, and hepatic fibrosis). Previous HBV/HCV infection with virus clearance was not counted as a high-risk factor by current guidelines [2]. All the clinical data and tumor imaging characteristics were collected at the time of diagnosis. Histopathological diagnosis of liver nodule was identified by regular pathological reports. If liver nodule was reported negative or benign, it was required to perform a second pathological confirmation or a follow-up without imaging changes for at least 12 months [14]. Cirrhosis was determined according to the histopathological Kleiner classification(≥ F4) of the liver tissue[15]. If the information of hepatic fibrosis was not available, cirrhosis was determined by unequivocal clinical manifestation of decompensated cirrhosis (like ascites or esophageal varices) or by radiographic features (like liver lobe imbalance or hepatic unevenness) [16, 17].

### Imaging evaluation

Imaging of candidate liver nodule was reviewed by two abdominal radiologists separately (ST.F., PZ.P. Both with over 20 years of experience in abdominal radiology). Both radiologists were blinded to pathological and clinical information. All the major imaging features and ancillary imaging features were analysed by one of two radiologists. The major imaging features include non-rim enhancement, washout and capsule enhancement. Table S3 showed the categories of ancillary imaging features. The benchmark and level of grading can be adjusted according to LI-RADS v2018. In cases of inconsistency, the assessments were jointly performed by both radiologists until they achieved an internal agreement [2]. Prior to the evaluation, the radiologists trained to interpreted fifty randomly selected computer tomogram (CT) or magnetic resonance imaging (MRI) strictly according to the LI-RADS v2018 to reduce their inter-rater reliability [18]. For patients with multiple nodules, we determined the target nodule for further assessment on imaging based on location information provided by surgical records, puncture records and pathology reports. Multifocal patients were excluded if none of lesion location was matched.

### MR imaging acquisition

MRI examination was performed by using a 3.0-T system (Magnetom trio, Siemens Healthineers, Erlangen, Germany). The scanning was ranging from the diaphragmatic crest to the anterior superior iliac spine. Eight-channel phased array coil was used and the MR sequence included: half-fourier single-shot turbo spin-echo (HASTE) sequence, fast low angle shot (FLASH) T1WI in/out of phase sequence imaging, FLASH T2WI fat suppression (FS) sequence axial imaging, and turbo spin-echo (TSE) T2WI navigation trigger axial imaging, Diffusion-weighted images (DWI) spin-echo plane sequence imaging (B = 0 and 800 s/mm2). After as bolus injection of Gd-EOB-DTPA (Primovist®,0.1 mL/kg) with a flow rate of 1 mL/s or with Omniscan (extracellular contrast agent, 0.1 mmol/kg) at a flow rate of 3 ml/s. The images in Arterial phase (20–25 s) portal-venous phase (65–70 s) and hepatic venous phase (100–120 s) were performed respectively. Additional transitional phase and hepatobiliary phase images were obtained from hepatobiliary contrast agent MRI (HBA-MRI) at 15 min and 20 min after injection. The selection of MRI contrast agent was based on the clinical practice.

### CT imaging acquisition

64 slice spiral CT machine (Aquilion One, Toshiba Medical System, Tokyo, Japan) was used for dynamic contrast-enhanced hepatic CT scans in both institutions. the scanning parameters were as follows. 120 kVp, 200 mAs tube voltage or 250 mA (using automatic tube current modulation) tube current, 64 × 0.5 mm detector collimating, 1 or 1.375 spacing, 10 mm thick slices layer and 0.5 s rotation time of the gantry. The peripheral intravenous iohexol (Ultravist, Bayer, Germany) 70 ml (at the rate of 3.0 ml/s), and 30 ml saline flushing after plain abdominal CT performed. Enhanced scan (arterial phase 20-25 s, portal phase 65-70 s, delayed phase 85-90 s) was performed from the diaphragmatic crest to the anterior superior iliac spine, covering the entire liver area.

### Statistical analysis

We split the consecutive patients from the 1st institution into two cohorts based on the time of admission. The former 70% patients constituted the training cohort (*n* = 325) and the rest constituted the internal validation sets (*n* = 138). All patients from the 2nd institution were served as the external validation cohort (*n* = 218).

Categorical variables are expressed as numbers and percentages. Continuous variables are showed as median and interquartile range (IQR). Comparisons between groups were performed using Kruskal–Wallis test for quantitative data and the Chi-square test or Fisher’s exact test for qualitative data. Cohen’s ĸ statistics were used for the evaluation of interobserver agreement. Logistic regression models were used to predict the risk of HCC diagnosis. Imaging variables with *p* < 0.05 on univariate analysis were included into multivariate regression analysis (forward stepwise with *p* < 0.01) to build the imaging model for HCC diagnosis based on the training cohort. When adding clinical variables, the clinical model was also constructed by following the same procedure. The risk score of each selected feature was based on the β coefficient in multivariable analysis. To assess the predictive performance of the models, relative operating characteristic curve (ROC curve) was plotted and AUC value in each cohort was calculated. Pairwise comparison of AUC values was achieved using the DeLong test. In order to avoid biopsy, non-invasive diagnostic criteria need to be weighted toward a very high specificity. Therefore, the cut-off values of each model were determined as guidelines recommended that diagnostic specificity is higher than 95% for definitive diagnosis of HCC [19]. The sensitivity, specificity, positive predictive value (PPV), and negative predictive value (NPV) of the prediction models were identified at each cohort. A risk score was calculated by summing up the coefficients of feature in multivariate regression analysis. We illustrated all the possible combinations of selected features to diagnose HCC if the risk score was higher than the cut-off threshold. Then all the features under each diagnostic combination were integrated and reconciled with clinical judgment. A two-sided *P* value < 0.05 is considered as a statistically significant difference. Statistical analysis was undertaken using R software version 3.6.1.

## Results

### Study population

Total of 681 patients in 2 institutions were included (shown in Table S1). Among them, 463 patients were enrolled from the 1st institution and 218 patients were from the 2nd institution. Median ages were between 53–60 years and over 50% of patients were male (58%-68%); Of these patients, the number of HCC are 174 (37.6%) and 103 (48%) in the two institutions, respectively (shown in Table S1). Of the remaining non-HCC patients, there were 170 and 47 cases of malignancies in the 1st institution and the 2nd institution. Predominant causes are iCCA, mixed HCC-CC and sarcoma. The rest were diagnosed with benign, most common types are FNH, inflammatory pseudotumor, angioleio-myolipoma and liver abscess (shown in Table S2). Table 1 showed the specific demographic and clinical characteristics of the training cohort, internal validation cohort and external cohort. Nearly 80% of nodules exceed 3 cm. There were significant differences among the three cohorts in age, family history, cigarette, alcohol, cardiovascular disease, HBcAb seropositive, HbeAb seropositive, AST, ALP, GGT, CA125, CA19-9 (all *p* < 0.05).

**Table 1:** Baseline demographics and clinical characteristics of the study population in different cohort

Characteristics	Train cohort (*n* = 324)	Internal validation cohort (*n* = 139)	External validation cohort (*n* = 218)	*P* value
Age (y, median, IQR)	59 (49, 66)	60 (50, 67)	53 (44, 68)	0.001^a^
Male (n, %)	189 (58%)	90 (65%)	149 (68%)	0.054
HCC (n, %)	112 (35%)	62 (45%)	103 (48%)	0.006
Family history (n, %)	11 (3%)	3 (2%)	20 (9%)	0.004
Cigarette (n, %)	69 (21%)	33 (24%)	57 (26%)	0.012
Alcohol (n, %)	42 (13%)	31 (22%)	57 (26%)	< 0.001
Cardiovascular disease (n, %)	67 (21%)	44 (32%)	33 (15%)	0.001
Diabetes (n, %)	39 (12%)	21 (15%)	27 (12%)	0.683
HCV (n, %)	3 (1%)	7 (1%)	2 (1%)	1.000
HBeAb + (n, %)	104 (32%)	58 (42%)	94 (43%)	0.018
HBcAb + (n, %)	216 (67%)	58 (42%)	94 (43%)	0.039
Platelet (median, IQR)	238 (184, 304)	227 (181, 293)	237 (194, 285)	0.629^a^
ALT (median, IQR)	25 (17, 49)	23 (15, 39)	24 (16, 35)	0.062^a^
AST (median, IQR)	30 (21, 51)	27 (20, 44)	22 (18, 30)	< 0.001^a^
GGT (median, IQR)	86 (46, 203)	76 (36, 175)	46 (28, 82)	< 0.001^a^
ALP (median, IQR)	107 (77, 186)	92 (72, 142)	85 (66, 111)	< 0.001^a^
AFP > 20 ng/L (n, %)	68 (21%)	32 (23%)	57 (26%)	0.374
CEA > 5ug/L (n, %)	62 (19%)	25 (18%)	23 (11%)	0.020
CA125 > 35U/ml (n, %)	81 (25%)	31 (22%)	3 (1%)	< 0.001
CA19-9 > 129U/ml(n, %)	66 (20%)	32 (23%)	13 (6%)	< 0.001
Size > 3 cm (n, %)	259 (80%)	116 (83.5%)	117 (81.2%)	0.697

The cutoff values of CEA, CA125 and CA19-9 are the upper limit of normal range

Abbreviations: *HCC* hepatocellular carcinoma, *AFP* alpha fetoprotein, *BMI* body mass index, *ALT* alanine transaminase, *AST* aspartate aminotransferase, *GGT* gamma-glutamyl transpeptidase, *ALP* alkaline phosphatase. *SD* standard deviation

^a^The data does not follow the normal distribution

### Diagnostic performance of major features for HCC in low-risk population

All the three major features of HCC had a similar accuracy (ACC, 73.0%-78.0%). Non-rim APHE is of a sensitivity of 79.9% and a specificity of 76.8% respectively. Non-peripheral washout achieved a comparable sensitivity of 86.8% and a moderate specificity of 64.7% to the non-rim APHE. Enhancing capsule achieved a low sensitivity of 39.7% but a high specificity of 96.9% (shown in Table 2). Consistency of imaging diagnosis between the 2 radiologists was robust(κ = 0.86, 95% CI: 0.75, 0.97).

**Table 2:** Diagnostic performance of major features for HCC diagnosis

Major features for HCC	Accuracy % (95% CI)	Sensitivity % (95% CI)	Specificity % (95% CI)	PPV % (95% CI)	NPV % (95% CI)
Non-rim APHE	78.0 (73.9–81.7)	79.9 (73.2–85.6)	76.8 (71.5–81.6)	67.5 (60.6–73.8)	86.4 (81.6–90.3)
Non-peripheral washout	73.0 (68.7–77.0)	86.8 (80.8–91.4)	64.7 (58.9–70.2)	59.7 (53.4–65.8)	89.0 (84.0–92.9)
Enhancing capsule	75.4 (71.2–79.2)	39.7 (32.3–47.3)	96.9 (94.2–98.6)	88.5 (79.2–94.6)	72.7 (68.0–77.1)

Abbreviations: *HCC* hepatocellular carcinoma, *CI* confidence interval, *APHE* arterial hyperenhancement, *PPV* positive predictive value, *NPV* negative predictive value

Table S3 summarized the diagnostic performance of ancillary features. The ancillary imaging features had ACCs with a range of 61.6%-71.9% to diagnose HCC and the mosaic architecture achieved the highest ACC (79%, 95%CI 67.6%–76.0%). Significantly, the specificities of ancillary features favoring HCC in particular were satisfying (86.9–99.3%). As for malignancy diagnosis, the sensitivity of ancillary features ranged from 2.9% to 100.0% and the highest was mild-moderate T2 hyperintensity (100%, 95%CI 92.6%–100.0%). The specificity of ancillary features in favor of malignancy ranged from 12.8% to 99.7% and the highest was fat sparing in solid mass (98.8%, 95%CI 96.9%–99.7%).

### Model-based prediction of HCC diagnosis in low-risk population

Univariate logistic regression analysis was performed in the training cohort (shown in Table S4). In multivariate logistic regression analysis only based on imaging features, the factors significantly associated with diagnosis of HCC were non-rim APHE (odds ratio (OR) 6.73, 95% CI 2.69–16.84), WO (OR 6.61, 95% CI 2.44–17.95), enchancing capsule (OR 9.37, 95% CI 2.83–31.07), tumor necrosis or severe ischemia (TNSI, OR 3.92, 95% CI 1.60–9.56) All the factors above were chosen to build the imaging model to diagnose HCC (shown in Table 3). The presence of TNSI was carefully identified as the area where it showed unenhanced and hypointensity within the nodule through plain scan and all enhanced phases. Figure S1-2 showed an example of TNSI within a typical HCC, atypical HCC by both readers.

**Table 3:** Multivariate logistic regression analysis including the imaging and clinical features statistically significant

Criteria	Odds ratio (95% CI)	β coefficient	*P* value
Imaging and clinical characteristics			
Non-rim APHE	6.73 (2.69, 16.84)	1.91 (0.99, 2.82)	< 0.001
Washout	6.61 (2.44, 17.95)	1.89 (0.89, 2.89)	0.001
Enhancing capsule	9.37 (2.83, 31.07)	2.24 (1.04, 3.44)	< 0.001
TNSI	3.92 (1.60, 9.56)	1.36 (0.47, 2.26)	0.003
AFP ≥ 20 ng/ml	39.68 (12.38, 127.17)	3.68 (2.52, 4.85)	< 0.001
Sex (male)	4.55 (1.85, 11.12)	1.52 (0.61, 2.42)	0.001
Imaging characteristics			
Non-rim APHE	7.22 (3.46, 15.07)	1.98 (1.24, 2.71)	< 0.001
Washout	7.17 (3.33, 15.43)	1.97 (1.20, 2.74)	< 0.001
Enhancing capsule	9.70 (3.62, 25.93)	2.27 (1.29, 3.26)	< 0.001
TNSI	5.45 (2.61, 11.36)	1.70 (0.96, 2.43)	< 0.001

Abbreviations: *CI* confidence interval, *APHE* arterial hyperenhancement, *AFP* alpha fetoprotein, *TNSI* Tumor necrosis or severe ischemia

When adding the clinical characteristics into multivariate logistic regression analysis, the same imaging characteristics above and two more clinical characteristics, alpha fetoprotein (AFP) > 20 ng/ml (OR 39.68, 95% CI 12.38–127.17) and male (OR 4.55, 95% CI 1.85–11.12) were independent risk factors for diagnosis of HCC and were utilised to build the clinical model (Table 3). The functions are exhibited in the supplementary material. The next step was to develop an HCC-risk score of liver nodule based on a points system and to simplify the computation procedure. The final score of the nodule was added by the corresponding risk scores of each feature which is exhibited in the nodule on imaging. The clinical model achieved the best performance with an AUC of 0.954 (95%CI 0.930–0.978), followed by imaging model (AUC = 0900, 95%CI 0.863–0.936) in training cohort.

### Validating the prediction models between the training and validation cohorts

**Fig. 2:**
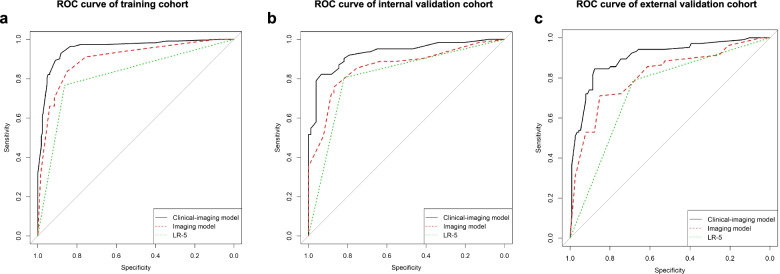
Receiver operating characteristic (ROC) curves of LR-5 (definite HCC based on LI-RADS v2018, green color), Imaging model (red color) and Clinical (black color) performed in training cohort (**a**), internal cohort (**b**) and external cohort (**c**)

Further, we validated the models both in the internal cohort and in the external validation cohort. The clinical model obtained an AUC of 0.931 (95%CI 0.886–0.975) in the internal validation cohort and 0.902 (95%CI 0.860–0.944) in the external validation cohort, respectively. The AUC of imaging model were 0.859 (95%CI 0.793–0.924) in the internal validation cohort and 0.813 (95%CI 0.755–0.871) in the external validation cohort, respectively (shown in Table 4). ROC curve further demonstrated that the clinical model has the highest accuracy for HCC diagnosis in the training cohort, the internal cohort and the external cohort (shown in Fig. 2).

**Table 4:** Diagnostic performance of LI-RADS v2018 and non-invasive model in each cohort

	Model	AUC % (95% CI)	ACC (no.)	SEN (no.)	SPE (no.)	PPV (no.)	NPV (no.)
Training cohort *n* = 324	LI-RAD v2018	81.6 (77.0–86.1)	83.0 (269/324)	76.8 (86/112)	86.3 (183/212)	74.8 (86/115)	87.6 (183/209)
	Imaging model	90.0 (86.3–93.6)	75.3 (244/324)	31.3 (35/112)	98.6 (209/212)	92.1 (35/38)	73.1 (209/286)
	Clinical model	95.4 (93.0–97.8)	89.2 (289/324)	77.7 (87/112)	95.3 (202/212)	89.7 (87/97)	89.0 (202/227)
Internal validation cohort *n* = 139	LI-RAD v2018	81.2 (74.6–87.8)	81.3 (113/139)	80.7 (50/62)	81.8 (63/77)	78.1 (50/64)	84 (63/75)
	Imaging model	85.9 (79.3–92.4)	74.1 (103/139)	51.6 (32/62)	92.2 (71/77)	84.2 (32/38)	70.3 (71/101)
	Clinical model	93.1 (88.6–97.5)	88.5 (123/139)	79.0 (49/62)	96.1 (74/77)	94.2 (49/52)	85.1 (74/87)
External validation cohort *n* = 218	LI-RAD v2018	73.6 (67.8–79.4)	73.4 (160/218)	78.9 (82/104)	68.4 (78/114)	69.5 (82/118)	78.0 (78/100)
	Imaging model	81.3 (75.5–87.1)	71.6 (156/218)	53.9 (56/104)	87.7 (100/114)	80.0 (56/70)	67.6 (100/148)
	Clinical model	90.2 (86.0–94.4)	85.3 (186/218)	81.7 (85/104)	88.6 (101/114)	86.7 (85/98)	84.2 (101/120)

Abbreviations: *HCC* hepatocellular carcinoma, *PPV* positive predictive value, *NPV* negative predictive value, *CI* confidence intervals, *ACC* accuracy, *AUC* area under curve, *SEN* sensitivity, *SPE* specificity

To achieved high specificity over 95%, the cut-off value was set to be 6.67 according to the training cohort. Under the cut-off value of 6.67, the sensitivity, specificity, PPV and NPV of the clinical model are 77.7%, 95.3%, 89.7%, 89.0% in the training cohort, 79.0%, 96.1%, 94.2%, 85.1% in the internal validation cohort and 81.7%, and 88.6%, 86.7%, 84.2% in the external validation cohort, respectively. The clinical model provided much higher sensitivity but similar specificity than that of the imaging model in each cohort (all *p* < 0.01, shown in Table 4). Considering there are multiple imaging modalities included in this study, we also observed the performance of the models in CT, ACE-MRI, and HBA-MRI subgroups. The diagnostic efficacies are similar with AUC values of 0.886 (95%CI 0.814–0.959), 0.913 (95%CI 0.857–0.968), and 0.893 (95%CI 0.781–0.100) respectively (shown in Table S5).

**Fig. 3:**
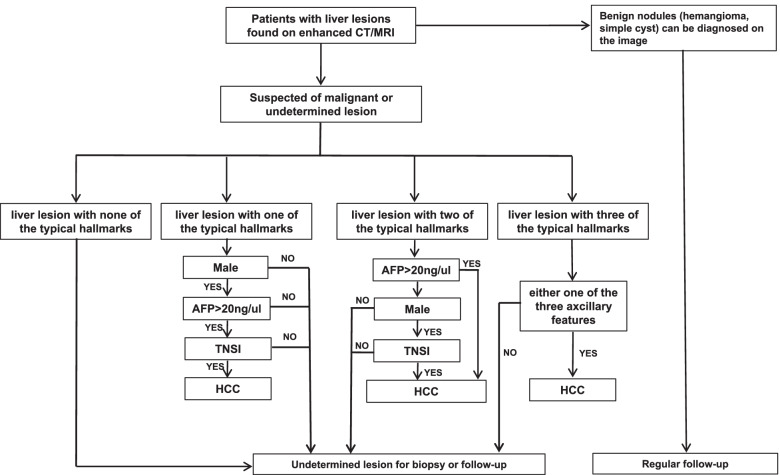
The diagnostic flow of primary liver nodules in low-risk population. Each scenario to diagnose HCC depends on the number of major features of HCC. The major features are hyperenhancement (APHE), wash-out (WO) and enchancing capsule. I). If a liver nodule is with only one of the HCC major features, then all three ancillary features are needed to diagnose HCC. II). If a liver nodule meets two of the major features, HCC could be diagnosed by either a male patient with TNSI in nodule or a patient with AFP > 20 ng/ml. III). If a liver nodule meets all of the major features, HCC could be diagnosed under the circumstance where one of ancillary features is satisfied

For more convenient application of the model in clinical practice, we calculated the risk score of each feature by the corresponding β coefficient in regression function. Then, we arranged and combined with risk features where the cumulative risk score was higher than the cut-off value. Finally, we listed all the clinical scenarios to diagnose HCC and made slight adjustments according to the routine diagnostic procedures of clinical experts and imaging experts. We made a simple flow diagram to diagnose HCC under each clinical scenario in LRP (shown in Fig. 3). First, different algorithms for the diagnosis of HCC depend on the number of major features of HCC presented in the target nodule. For example, when a liver nodule has only one of the three major features of HCC, all of the ancillary features (male gender, AFP > 20 ng/ml, and TNSI) must be met to diagnose HCC, and if a liver nodule has met all of the three major features, only one of the ancillary features is needed to diagnose HCC.

## Discussion

In addition to APHE, WO, enhancing capsule, we found that ancillary imaging feature TNSI, along with clinical features including male gender and elevated AFP also supported diagnosis of HCC. Accordingly, we established a satisfactory HCC non-invasive diagnostic model for LRP with high specificity, especially constructed by both relevant clinical and imaging features.

To our knowledge, this study firstly reported the imaging diagnostic performance in a cohort study. A case–control study conducted by Ludwig DR et al. suggested high specificity using LI-RADS v2018 to distinguish HCC from non-HCC primary liver malignancy. However, their study excluded benign nodules so as to inaccurately evaluate the real diagnostic performance of imaging procedure in LRP [20]. Although current imaging criteria with over 95% specificity to diagnose HCC larger than 10 mm have been verified in HRP, we found that it was not qualified for LRP owing to its significant lower specificity and PPV. Considering a total of 74 nodules were misdiagnosed as HCC and the benign are taken up over 60%. Therefore, we do not recommend using LI-RADS criteria to diagnose HCC in LRP.

In fact, all the HCC major features also present on a few of non-HCC nodules such as iCCA, adenoma, Angioleiomyolipoma et al., which are more common in LRP [6, 21]. HCC usually takes up over 90% in HRP while it only accounts for 40.7% in LRP according to our results [22]. We speculated that epidemiological difference between these two population greatly contributes to the inconsistent diagnostic efficacy. To break the bottleneck, apart from the HCC major features, this evidence-based model incorporates ancillary imaging features and clinical features as required to definitely diagnose HCC with high specificity. Of these features, TNSI as an LR-M ancillary feature presents in around 70% of malignancies while only in 30% of benign in all cohorts (our data has not shown). Although the specificity of TNSI in distinguishing HCC from malignant nodules is weak (TNSI is present in 75% of HCC), this feature still could tell apart from benign nodules in LRP. Previous studies demonstrated that HCC in the non-cirrhotic patients was common with certain areas of necrosis. One possible explanation is that the mean size of nodules is greater than 5 cm in this study. Excessive tumor growth beyond the blood supply capacity leads to hypoxia and might make intra-tumoral necrosis more common. [8, 23].

Our study indicated that gender greatly impacts on diagnostic algorithm in LRP. Male are more likely to develop HCC than female and many HCC-like nodules such as focal nodular hyperplasia (FNH) and hepatocellular adenoma occur principally in young and middle-aged women in LRP, especially those who have taken oral contraceptive clinically [24]. Of note, AFP as a consensus risk factor, elevated value not only indicates HCC development but activate hepatitis or cirrhosis. Consequently, its diagnostic specificity is not high in HRP. Fortunately, such conditions seem less likely to occur in LRP. To the end, we identified 20 ng/ml as a cutoff value for its good diagnostic sensitivity without weakening the specificity [25].

The diagnostic performance of clinical models achieved good performance both in the training cohort and in the internal validation cohort. However, we noticed that the diagnostic efficacies of both models in the external validation were generally lower. The diversities of demographic and clinical characteristics among our cohorts might lead to the nuance of diagnostic efficacy. Since all of the patients in the external validation cohort underwent surgery and were younger than the other two cohorts. Besides, we also demonstrated that the diagnostic performances of our model were satisfactory in the CT, ACE-MRI, and HBA-MRI subgroup and were comparable to the performances in HRP previously reported (Table S5, *p* > 0.05) [19]. This result indicated that a widely potential application of this model is feasible in multiple examinations. It is worth noting that although there are some differences in diagnostic sensitivity and specificity of the imaging features depending on indivudual examination for example, the sensitivity of enhancing capsule on MRI is much higher than that on enhanced CT, the common features in different examination are combined to be analysed in this study as the same principle of LI-RADS was performed in HRP. However, HBA-MRI is generally considered to have a higher accuracy in diagnosis of HCC which did not exhibit in this study possibly due to the small sample size. A further validation is needed (Table S5).

In general, our diagnostic algorism does not require clinicians to perform complex calculations, but rather a simple flowchart to diagnose HCC (Fig. 3). Briefly, if a liver nodule cannot definitely be diagnosed in LRP, the clinicians could firstly confirm the major features of HCC the nodule characterizes, and then analyse the specific ancillary features (including TNSI, elevated APF, and male gender) to diagnose HCC according to the number of major features the nodules satisfied. If the liver nodule are still indeterminate, a biopsy was recommended for further confirmation. Of note, we did not include the liver metastasis in the study because we thought that the diagnostic strategy of suspected metastasis was different from primary liver nodules. These patients usually have extrahepatic symptoms of primary diseases and the diagnostic sensitivity should be given priority. Therefore, this model should be applied as an ancillary diagnostic approach of HCC and the final diagnosis depends on the comprehensive judgements of clinicians.

This study had some limitations. We were aware that selection bias was inevitable during the patient collection. This study only included patients with definite pathological information which was not fully representative of the LRP. Considering this situation, our model is more applicable for clinicians to assist in the diagnosis of highly suspected patients, not as a screening strategy in LRP. However, the majority of the patients included in this study was in the highlight that clinicians considered worthy of taking a biopsy. Hence, the risk features in this study require more attention from clinicians. Moreover, because of the retrospective data missing, especially in the biopsy reports, the non-invasive criteria of cirrhosis are adopted by clinical definitions which is a certain degree of subjectivity. Finally, limited cases of MRI examination restricted on further subgroup analysis of MRI parameters such as DWI and HBP.

## Conclusion

In conclusion, this is the first cohort study on non-invasive diagnosis of HCC in LRP. The clinical model we developed achieved a satisfactory non-invasive diagnostic performance in LRP and should be further investigated.

## Supplementary Information

**Additional file 1: Supplementary Figure 1.** HBA-MR images of a 55-year-old man in low-risk population with an HCC being classified to LR-5 (definite HCC). Pathological histology showed that HCC was with Edmondson II grade and its size was 6.9×5.1×4.2cm, liver fibrosis degree was F2. AFP was 3.33ng/ml.T1-weighted (a), T2-weighted (b), DWI (c), T2-weighted fat suppressed (i) phase showed a nodule with a lesion in hepatic segment I respectively. The nodule showed non-rim hyperenhancement in the arterial phase (d) and non-peripheral washout and enhancing capsule in the portal venous phase (e), venous phase (f),transitional phase (g) and hepatobiliary phase (h). The white arrow showed the necrotic area where it was unenhanced and hypointense during each phase.

**Additional file 2: Supplementary Figure 2.** HBA-MR images of a 63-year-old man in low-risk population with an HCC being classified to LR-M (definite malignancy). HCC is with Edmondson II grade and its size was 6×5×4cm. Liver fibrosis degree was F2. AFP was 3.98ng/ml.T1-weighted (a) and T2-weighted (b) phase shows a nodule with haemorrhage sign in hepatic segment VI/VII. A rim hyperenhancement and delayed central enhancement with tumor necrosis show in (d) the portal venous phase, venousphase (e) and transitional phase (g). Lesion shows hypointense in hepatobiliary phase (h). T2-weighted fat suppressed unenhanced MR image (i) and DWI (c) also showed a hyperintense lesion. The white asterisk showed the hyperintense area in plain scan where it thought to be intra-tumorous hemorrhage. The white arrow showed the necrotic area where it was unenhanced and hypointense during each phase.

**Additional file 3: SupplementaryTable 1.** Patients and nodules characteristics of institution 1 and 2. **Supplementary Table 2. **The number of each liver lesion according to pathological classification. **Supplementary Table 3.** Performance ofancillary features for HCC diagnosis. **SupplementTable 4.** Univariate analysis of baseline characteristics with *P*<0.05 on diagnosis for HCC. **Supplement Table 5.**Diagnostic performance of LI-RADS v2018 based on different radiological examination in the external cohort.

## Data Availability

All data generated or analysed during this study are included in this published article and its supplementary information files.

## Abbreviations

HCC: Hepatocellular carcinoma
LRP: Low-risk population
HRP: High-risk population
NAFLD: Non-alcoholic fatty liver disease
CHB: Chronic hepatitis B
APHE: Hyperenhancement in the arterial phase
WO: Wash-out
AFP: Alpha fetoprotein
LI-RADS: Liver Imaging Reporting and Data System
CT: Computed tomography
MRI: Magnetic resonance imaging
DWI: Diffusion-weighted images
HBA-MRI: Hepatobiliary contrast agent MRI
NPV: Negative predictive value
OR: Odds ratio
PPV: Positive predictive value
ROC curve: Relative operating characteristic curve
AST: Aspartate aminotransferase
ALP: Alkaline phosphatase
ACC: Accuracy
iCCA: Intrahepatic cholangiocarcinoma
TNSI: Tumor necrosis and severe ischemia
FNH: Focal nodular hyperplasia
