# Supplementary figures and images for: Non-invasive diagnosis strategy of hepatocellular carcinoma in low-risk population
Source: BMC Cancer. 2022 Jun 28;22:709. doi: 10.1186/s12885-022-09812-w (PMC9238050; doi:10.1186/s12885-022-09812-w)

## Slide 1
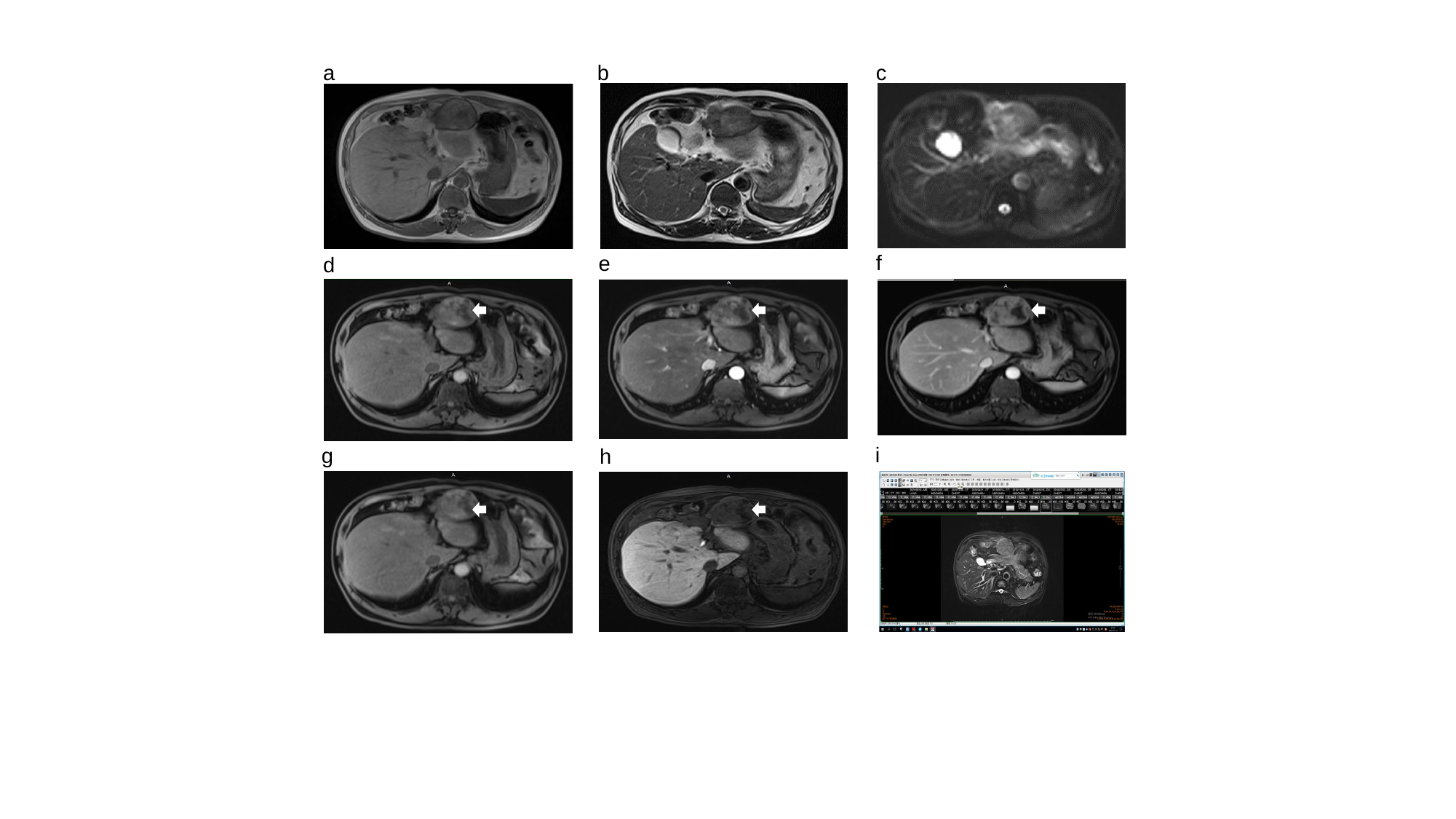

a
c
b
f
e
d
i
g
h

Supplement: Supplementary file 1 — Additional file 1: Supplementary Figure 1. HBA-MR images of a 55-year-old man in low-risk population with an HCC being classified to LR-5 (definite HCC). Pathological histology showed that HCC was with Edmondson II grade and its size was 6.9×5.1×4.2cm, liver fibrosis degree was F2. AFP was 3.33ng/ml.T1-weighted (a), T2-weighted (b), DWI (c), T2-weighted fat suppressed (i) phase showed a nodule with a lesion in hepatic segment I respectively. The nodule showed non-rim hyperenhancement in the arterial phase (d) and non-peripheral washout and enhancing capsule in the portal venous phase (e), venous phase (f),transitional phase (g) and hepatobiliary phase (h). The white arrow showed the necrotic area where it was unenhanced and hypointense during each phase. [file 12885_2022_9812_MOESM1_ESM.pptx]

## Slide 1
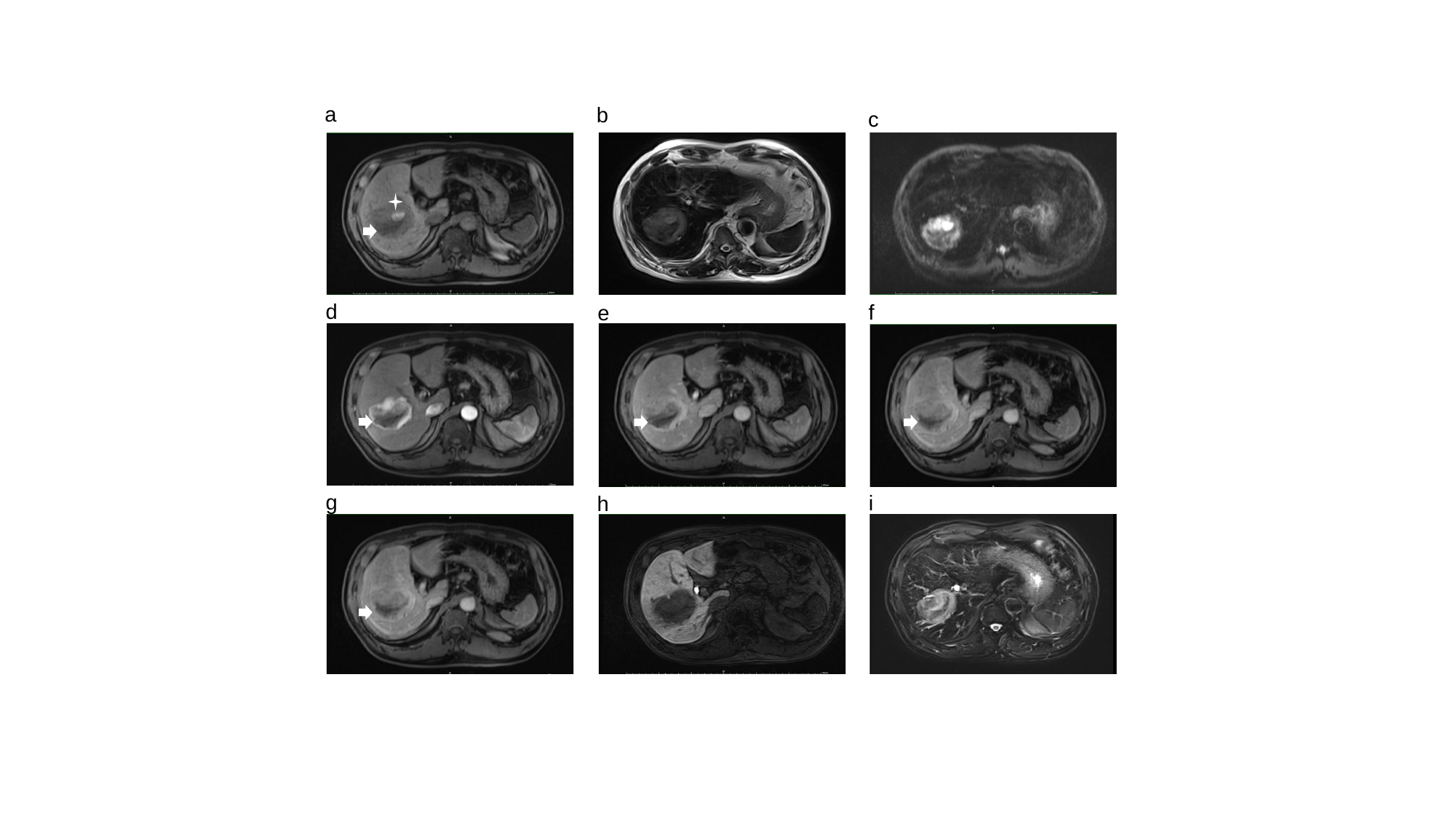

a
b
c
d
f
e
g
i
h

Supplement: Supplementary file 2 — Additional file 2: Supplementary Figure 2. HBA-MR images of a 63-year-old man in low-risk population with an HCC being classified to LR-M (definite malignancy). HCC is with Edmondson II grade and its size was 6×5×4cm. Liver fibrosis degree was F2. AFP was 3.98ng/ml.T1-weighted (a) and T2-weighted (b) phase shows a nodule with haemorrhage sign in hepatic segment VI/VII. A rim hyperenhancement and delayed central enhancement with tumor necrosis show in (d) the portal venous phase, venousphase (e) and transitional phase (g). Lesion shows hypointense in hepatobiliary phase (h). T2-weighted fat suppressed unenhanced MR image (i) and DWI (c) also showed a hyperintense lesion. The white asterisk showed the hyperintense area in plain scan where it thought to be intra-tumorous hemorrhage. The white arrow showed the necrotic area where it was unenhanced and hypointense during each phase. [file 12885_2022_9812_MOESM2_ESM.pptx]
