# Supplementary material for: Non-invasive diagnosis strategy of hepatocellular carcinoma in low-risk population
Source: BMC Cancer. 2022 Jun 28;22:709. doi: 10.1186/s12885-022-09812-w (PMC9238050; doi:10.1186/s12885-022-09812-w)
Supplement: Supplementary file 3 — Additional file 3: SupplementaryTable 1. Patients and nodules characteristics of institution 1 and 2. Supplementary Table 2. The number of each liver lesion according to pathological classification. Supplementary Table 3. Performance ofancillary features for HCC diagnosis. SupplementTable 4. Univariate analysis of baseline characteristics with P<0.05 on diagnosis for HCC. Supplement Table 5.Diagnostic performance of LI-RADS v2018 based on different radiological examination in the external cohort. [file 12885_2022_9812_MOESM3_ESM.docx]

**Online-only Supplemental Data Content**

**Statement:** The Supplemental content are in the form originally provided by the authors.

Supplemental material

Supplementary Table 1 Patients and nodules characteristics of institution 1 and 2

Supplementary Table 2 The number of each liver lesion according to pathological classification.

Supplementary Table 3 Performance of ancillary features for HCC diagnosis.

Supplement Table 4. Univariate analysis of baseline characteristics with P<0.05 on diagnosis for HCC.

Supplement Table 5. Diagnostic performance of LI-RADS v2018 based on different radiological examination in external cohort.

**Supplemental material:**

The multivariate logistic functions were as follow:

$$predict probability=\frac{1}{1+e^{g(x)}}$$

**Imaging model**

$$g\left( x \right)=1.977884\times Nonrim APHE+1.969488\times WO+2.271952\times Capsule enhancement+1.696565\times necrosis-4.299675$$

**Clinical-imaging model**

$$g\left( x \right)=1.517822\times male+3.680762 \times AFP+1.905865\times Nonrim APHE+1.889261\times WO+2.237455\times capsule enhancement+1.364949 \times necrosis-5.788444$$

**Supplement Table:**

**Supplement Table1.Patients and nodules characteristics of institution 1 and 2.**

| Characteristics | Total  (n=681) | Institution 1  (n=463) | Institution 2  (n=218) | *P* value |
| --- | --- | --- | --- | --- |
| Patient’s characteristics | | | | |
| Age (y, median, IQR) | 58.0(47.0, 65.0) | 59.0(49.0, 66.0) | 53.8(44.0, 62.8) | <0.001 |
| Male (n, %) | 428 (62.8%) | 279 (60.3%) | 149 (68.3%) | 0.042 |
| BMI (median, IQR) | 22.9(20.8, 24.9) | 22.8(20.7, 25.0) | 23.0(21.1, 24.7) | 0.414^a^ |
| Family history (n, %) | 34 (5.0%) | 14 (3.0%) | 20 (9.2%) | 0.001 |
| Cigarette (n, %) | 173 (25.4%) | 102 (22.0%) | 71 (32.6%) | 0.003 |
| Alcohol (n, %) | 130 (15.8%) | 46 (26.4%) | 27 (9.3%) | <0.001 |
| Cardiovascular disease (n, %) | 144 (21.1%) | 111 (24.0%) | 33 (15.1%) | 0.008 |
| Diabetes (n, %) | 87 (12.8%) | 60 (13.0%) | 27 (12.4%) | 0.834 |
| HCV (n, %) | 6 (0.9%) | 4 (2.3%) | 2 (0.9%) | 1.000 |
| HBeAb+ (n, %) | 162 (35.0%) | 84 (48.3%) | 78 (27.0%) | <0.001 |
| HBcAb+ (n, %) | 434 (63.7%) | 310 (67.0%) | 124(56.9%) | 0.011 |
| Platelet (median, IQR) | 237.0  (187.0, 293.0) | 245.6  (183.0, 298.3) | 237.0  (194.3, 285.0) | 0.946^a^ |
| ALT (median, IQR) | 25.0(16.0, 41.0) | 25.0(16.0, 44.0) | 23.8(16.2, 34.5) | 0.106^a^ |
| AST (median, IQR) | 26.0(19.7, 43.0) | 29.0(21.0, 50.0) | 22.2(18.0, 30.1) | <0.001^a^ |
| GGT (median, IQR) | 70.0(34.0,155.6) | 85.0(42.0, 200.0) | 45.9(27.6, 82.3) | <0.001^a^ |
| ALP (median, IQR) | 96.0 (73.0, 149.9) | 103.0(75.0, 173.0) | 84.7(66.0,111.2) | <0.001^a^ |
| AFP >20ng (n, %) | 157 (23.1%) | 100 (21.6%) | 57 (26.1%) | 0.189 |
| CEA (median, IQR) | 2.4 (1.5, 3.6) | 2.5(1.6, 3.9) | 1.9(1.0, 3.1) | <0.001^a^ |
| CA125 (median, IQR) | 16.8 (12.9, 22.0) | 16.8(10.9, 32.1) | 16.8(9.8,20.4) | 0.647^a^ |
| CA19-9(median, IQR) | 12.4 (5.8, 34.9) | 12.4(4.9, 57.4) | 13.2(7.0,23.5) | 0.418^a^ |
| Treatment | | | | <0.001 |
| Surgery (n, %) | 567 (83.2%) | 349 (75.4%) | 218 (100%) |  |
| Transplantation (n, %) | 7 (1.0%) | 7 (1.5%) | 0 (0%) |  |
| Biopsy (n, %) | 107 (15.7%) | 107 (23.1%) | 0 (0%) |  |
| Lesion characteristics | | | | |
| Size (median, IQR) | 5.6 (3.6, 8.3) | 5.8 (3.5, 8.6) | 5.2 (3.6, 7.9) | 0.086^a^ |
| Single lesion (n, %) | 549 (80.6%) | 357 (77.1%) | 192 (88.1%) | <0.001 |
| Examination method |  |  |  | <0.001 |
| Enhanced CT | 466 (68.4%) | 386 (83.4%) | 80 (36.7%) |  |
| ACE-MRI | 160 (23.5%) | 59 (12.7%) | 101 (46.3%) |  |
| HBA-MRI | 55 (8.1%) | 18 (3.9%) | 37 (17.0%) |  |

Abbreviations: HCC, hepatocellular carcinoma; AFP, alpha fetoprotein; BMI, body mass index; HCV, hepatitis C virus; ALT, alanine transaminase; AST, aspartate aminotransferase; GGT, gamma-glutamyl transpeptidase; ALP, alkaline phosphatase. SD: standard deviations

^a^The data does not follow the normal distribution.

**Supplement Table 2.** **The number of each liver lesion according to pathological classification.**

| Lesion | Institution 1 | Institution 2 | All |
| --- | --- | --- | --- |
| HCC | 174 | 103 | 277 |
| iCCA | 150 | 28 | 178 |
| Mixed  HCC-CC | 7 | 10 | 17 |
| Sarcoma | 7 | 5 | 12 |
| FNH^a^ | 16 | 34 | 50 |
| Hemangioendothelioma | 3 | 0 | 3 |
| Abscess ^a^ | 21 | 0 | 21 |
| Adenoma ^a^ | 8 | 3 | 11 |
| Cystadenoma ^a^ | 9 | 2 | 11 |
| Angioleio-  Myolipoma^a^ | 11 | 10 | 21 |
| Inflammatory pseudotumor^a^ | 15 | 1 | 16 |
| Granuloma ^a^ | 10 | 0 | 10 |
| Non-special  Benign^a^ | 26 | 17 | 43 |
| Non-special  malignancy | 6 | 5 | 11 |
| Total | 463 | 218 | 681 |

Abbreviations: HCC, hepatocellular carcinoma; iCCA, intrahepatic cholangiocarcinoma; FNH, focal nodular hyperplasia; Mixed HCC-CC, mixed hepatocellular cholangiocarcinoma

Note: Percentages are based on each pathological type of the lesions.

^a^ the definition of benign lesions and the remains defined malignancy.

**Supplement Table 3. Performance of ancillary features for HCC diagnosis.**

| Ancillary Features Favoring Malignancy in General | Accuracy %  (95% CI) | Sensitivity %  (95% CI) | Specificity %  (95% CI) | PPV %  (95% CI) | NPV %  (95% CI) |
| --- | --- | --- | --- | --- | --- |
| Corona enhancement | 61.3  (56.7-65.8) | 3.4  (1.3-7.4) | 96.2  (93.3-98.0) | 35.3  (14.2-61.7) | 62.3  (57.7-66.8) |
| Fat sparing in solid mass | 63.3  (58.7-67.7) | 2.9  (0.9-6.6) | 99.7  (98.1-100.0) | 83.3  (35.9-99.6) | 63.0  (58.4-67.5) |
| Mild-moderate T2 hyperintensity ^a^ | 56.4  (44.7-67.6) | 100.0  (92.6-100.0) | 12.8  (4.3-27.0) | 53.4  (41.4-65.2) | 100.0  (54.9-100.0) |
| Restricted diffusion^a^ | 70.5  (59.1-80.3) | 97.4  (86.5-99.9) | 43.6  (27.8-60.0) | 63.3  (49.9-75.4) | 94.4  (72.7-99.9) |
| Transitional phase hypointensity^b^ | 67.9  (56.4-78.1) | 79.5  (63.5-90.7) | 56.4  (39.6-72.0) | 64.6  (49.5-77.8) | 73.3  (54.1-87.7) |
| Hepatobiliary phase hypointensity^b^ | 65.7  (61.1-70.0) | 17.8  (12.4-24.3) | 94.5  (91.2-96.0) | 66.0  (50.7-79.1) | 65.6  (60.8-70.2) |
| Ancillary Features Favoring HCC in Particular | - | - | - | - | - |
| Non-enhancing capsule | 61.6  (57.0-66.0) | 0.00  (0.00-1.70) | 98.6  (96.5-99.6) | 0.00  (0.00-5.27) | 62.1  (57.5-66.5) |
| Mosaic architecture | 71.9  (67.6-76.0) | 47.1  (39.5-54.8) | 86.9  (82.4-90.5) | 68.3  (59.2-76.5) | 73.2  (68.2-77.8) |
| Fat in mass | 64.1  (59.6-68.5) | 9.80  (8.50-15.2) | 96.9  (94.2-98.6) | 65.4  (44.3-82.8) | 64.1  (59.4-68.6) |
| Blood products in mass | 65.9  (61.4-70.2) | 12.6  (8.10-18.5) | 97.9  (95.5-99.2) | 78.6  (59.0-91.7) | 65.1  (60.4-69.5) |
| Nodule-in-nodule architecture | 63.5  (58.9-67.9) | 4.00  (1.60-8.10) | 99.3  (97.5-99.9) | 77.8  (40.0-97.2) | 63.2  (58.6-67.7) |
| Major features Favoring Malignancy in Genera | - | - | - | - | - |
| Rim APHE | 35.2  (30.9-39.7) | 16.1  (11.0-22.4) | 46.7  (40.8-52.0) | 15.2  (10.5-21.5) | 48.0  (42.1-54.1) |
| Peripheral washout | 59.4  (54.8-63.9) | 6.9  (3.6-11.7) | 91.0  (87.1-94.0) | 31.6  (17.5-48.7) | 61.9  (57.1-66.5) |
| Delayed central enhancement | 57.9  (53.2-62.4) | 0.0  (0.0-1.7) | 92.7  (89.1-95.0) | 0.0  (0.0-1.3) | 60.6  (55.9-65.2) |
| Infiltrative appearance | 46.0  (41.4-50.7) | 25.9  (19.5-33.0) | 58.1  (52.2-63.0) | 27.1  (20.5-34.5) | 56.6  (50.7-62.3) |
| Necrosis or severe ischemia | 53.8  (49.1-58.4) | 75.9  (68.8-82.0) | 40.5  (34.8-46.0) | 43.4  (37.8-49.2) | 73.0  (66.0-80.3) |
| Targetoid TP or HBP^b^ | 47.4  (36.0-59.1) | 12.8  (4.3-27.4) | 82.1  (66.5-92.0) | 41.7  (15.2-72.3) | 48.5  (36.0-61.1) |
| Targetoid restriction^a^ | 39.7  (28.8-51.5) | 2.6  (0.1-13.5) | 76.9  (60.7-88.0) | 10.0  (0.3-44.5) | 44.1  (32.1-56.7) |

Abbreviations: HCC, hepatocellular carcinoma; CI, confidence intervals.

Note: ^a^ Data assessed in the HBA-MRI and ECA-MRI group, ^b^ Data assessed only in the HBA-MRI group.

**Supplement Table 4. Univariate analysis of baseline characteristics with P<0.05 on diagnosis for HCC.**

| Characteristics | Odds ratio  (95% CI) | P value | Characteristics | Odds ratio  (95% CI) | P value |
| --- | --- | --- | --- | --- | --- |
| Age | 1.02 (1.00,1.03) | 0.037 | Non-rim APHE  (yes vs. no) | 13.16 (8.30,20.85) | <0.001 |
| Sex  (female vs. male) | 0.24 (0.15,0.37) | <0.001 | Non-peripheral washout  (yes vs. no) | 12.04 (7.30,19.86) | <0.001 |
| Cigarette  (yes vs. no) | 2.64 (1.69,4.14) | <0.001 | Enhancing capsule  (yes vs. no) | 20.44 (9.85,42.42) | 0.004 |
| Alcohol  (yes vs. no) | 3.49 (2.07,5.86) | <0.001 | fat in mass  (yes vs. no) | 3.37 (1.47,7.73) | 0.004 |
| Diabetes  (yes vs. no) | 2.27 (1.31,3.93) | 0.003 | Mosaic architecture  (yes vs. no) | 6.01 (1.23,29.27) | 0.026 |
| HBeAb  (yes vs. no) | 2.52 (1.70,3.75) | <0.001 | Nodule-in-nodule  (yes vs. no) | 5.89 (3.74,9.26) | <0.001 |
| HBcAb  (yes vs. no) | 3.03 (1.94,4.74) | <0.001 | Infiltrative appearance  (yes vs. no) | 0.48 (0.32,0.73) | <0.001 |
| HCV  (yes vs. no) | 3.25 (1.27,8.32) | 0.014 | Blood products in mass  (yes vs. no) | 6.83 (2.71,17.20) | <0.001 |
| ALP | 1.00 (0.99,1.00) | <0.001 | Necrosis or severe ischemia  (yes vs. no) | 2.14 (1.41,3.25) | <0.001 |
| AFP  ≤20 vs >20 | 1.00 (1.00,1.00) | 0.003 | Rim APHE  (yes vs. no) | 0.17 (0.11,0.27) | <0.001 |
| CEA | 0.93 (0.89,0.98) | 0.007 | Transitional phase hypointensity  (yes vs. no) | 5.01 (1.84,13.66) | 0.002 |
| CA125 | 0.99 (0.99,1.00) | 0.004 | HBP hypointensity  (yes vs. no) | 5.57 (2.04,15.23) | <0.001 |
| CA19.9 | 1.00 (1.00,1.00) | 0.002 | Targetoid restriction  (yes vs. no) | 0.09 (0.01,0.73) | 0.025 |
|  |  |  | Restricted diffusion  (yes vs. no) | 29.36 (3.65,235.99) | 0.001 |

Abbreviations: HCC, hepatocellular carcinoma; CI, confidence intervals; AFP, alpha fetoprotein; HCV, hepatitis C virus; ALP, alkaline phosphatase;

CEA, carcinoma embryonic antigen; APHE, Arterial phase high enhancement; HBP, hepatobiliary phase

**Supplement Table 5.** **Diagnostic performance based on different radiological examination in external cohort.**

| Examination  (n) | Model | AUC  (95% CI) | Accuracy  (n) | Sensitivity  (n) | Specificity  (n) | PPV  (n) | NPV  (n) |
| --- | --- | --- | --- | --- | --- | --- | --- |
| CT (n=80) | Imaging model | 83.6  (75.0, 92.1) | 66.3  (59/89) | 31.0  (13/42) | 97.9  (46/47) | 92.9  (13/14) | 61.3  (46/75) |
|  | Clinical-imaging model | 88.6  (81.4, 95.9) | 84.3  (75/89) | 71.4  (30/42) | 95.7  (45/47) | 93.8  (30/32) | 79.0  (45/57) |
| ACE-MRI (n=101) | Imaging model | 83.7  (75.9, 91.6) | 71.4  (35/49) | 78.9  (41/52) | 76.1  (35/46) | 76.1  (35/46) | 74.6  (41/55) |
|  | Clinical-imaging model | 91.3  (85.7, 96.8) | 87.8  (43/49) | 80.8  (42/52) | 81.1  (43/53) | 81.1  (43/53) | 87.5  (42/48) |
| HBA-MRI  (n=37) | Imaging model | 70.2  (51.6, 88.7) | 47.1  (8/17) | 90.0  (18/20) | 80.0  (8/10) | 80.0  (8/10) | 66.7  (18/27) |
|  | Clinical-imaging model | 89.3  (78.1, 100.0) | 70.6  (12/17) | 95.0  (19/20) | 95.0  (19/20) | 92.3  (12/13) | 79.2  (19/24) |

Abbreviations: HCC, hepatocellular carcinoma; PPV, positive predictive value; NPV, negative predictive value; CI, confidence intervals; CT, computed tomography; HBA-MRI, hepatobiliary contrast agent magnetic resonance imaging; ACE-MRI: extracellular contrast agent magnetic resonance imaging
